# Carcinomata of the Gastro-Intestinal Tract1Read before the Buffalo Academy of Medicine, November 1, 1911.

**Published:** 1912-01

**Authors:** Wiliam J. Mayo

**Affiliations:** Rochester, Minn.


### BUFFALO MEDICAL JOURNAL

VOL. LXVII. JANUARY, 1912. No. 6

### ORIGINAL COMMUNICATIONS

### Carcinomata of the Gastro-Intestinal Tract[1]

By WILIAM J. MAYO, M.D.
Rochester, Minn.

FROM October 1, 1897 to November 1, 1911, 1264 cases of carcinomata of the gastro-intestinal tract were operated at St. Mary's Hospital. Of this number, 863 involved the stomach, 14 the small intestine, 219 the large intestine, and 168 the rectum. These statistics show that carcinomata of the stomach form more than two-thirds of all the carcinomata of the gastro-intestinal tract. Carcinomata of the large intestine come next in relative order, and the rectum follows with a smaller number.

In the total number (859) of carcinomata of the stomach, it was possible to do the radical operation in only 307, or 34.9 per cent. of the cases. In the group of 14 carcinomata of the small intestine, five, or 35.7 per cent. of the patients were submitted to radical operation; in carcinomata of the large intestine and rectum, nearly three-fourths of the patients were operated upon radically.

These data would indicate that the stomach is the organ most frequently affected with cancer, and that only one-third of the patients submitted to operation are operated with any prospect of cure. The balance are subjected to palliative operations, such as gastrojejunostomy, gastrostomy, or to explorations.

The number of carcinomata of the small intestine is curiously small compared with carcinoma of the associated organs. The small intestine is the most primitive part of the gastro-intestinal tract, and it is reasonable to suppose that its longer heredity makes it more resistant to malignancy than the stomach and large intestine, which organs were added later in the scheme of development.

In studying the question of permanent cures following resections of the gastro-intestinal tract, we find that, of the total number of cases of cancer of the stomach submitted to radical operation, more than 23 per cent. of the patients who recovered from the operation and whose present condition has been ascer-

1. Read before the Buffalo Academy of Medicine, November 1, 1911.

tained are alive and well over five years. Fifty per cent. of patients submitted to radical operation for cancer of the large intestine, and 30 per cent. with cancer of the rectum, who recovered from operation and whom we have been able to trace, are alive and well over five years. Every case dying within the 5 year period is counted as a death from the disease, which is not fair to the statistics since the normal death rate during the five year period would approximate 8 per cent.

Taken as a whole, these statistics indicate that carcinomata of the gastro-intestinal tract, which are sufficiently localized to justify radical operations, give results which are fully as good as operations for carcinomata in other parts of the body. The pessimism of the medical profession regarding malignant disease of the gastro-intestinal tract is not justified by the facts. It is the failure to make a diagnosis during the stage when the disease is still localized, and not any peculiar malignant tendencies of the process itself, which accounts for the fatal character of cancer in this region.

The most important question in connection with the radical cure of carcinomata concerns the lymphatics, and the proof of this assertion cannot be better shown than in the results as to permanent cure after resection of the various organs of the gastro-intestinal tract. The stomach is highly supplied with lymphatics and gives the smallest percentage of radical cures. The large intestine, having the least lymphatics, gives the highest percentage of radical cures, and the rectum, with a moderate supply of lymphatics, gives better results than the stomach, but less favorable results than the large intestine.

*The Stomach.* If one were to drop a line vertically from the cardiac orifice across the greater curvature, the stomach would be divided into two unequal parts, the larger part, composed of the dome and fundus, has but a scanty supply of lymphatics which pass to the left to the glands lying in the splenic area, and to the cardiac glands about the esophagus. The smaller plyoric segment, has nine-tenths of all the lymphatics of the stomach. They are divided into four groups: (1) The glands along the lesser curvature, lying in the wall of the stomach itself and draining into the glands about the celiac axis. (2) Those along the greater curvature which lie with the gastro-epiploic vessels. In this region the lymphatic current passes from left to right toward the pylorus. (3) That important group lying about the pylorus, the first portion of the duodenum, and the head of the pancreas. (4) The few glands which pass with the superior pyloric vessels. The situation of these four groups of glands is definitely determined by the four blood vessels, so that early ligation of the gastric, gastro-duodenal, superior pyloric, and gastro-epiploic vessels, not only permits bloodless operation but enables radical removal of the tributary lymphatics. Unfortunately, this glandular scheme of lymphatic drainage may be disturbed, first, because of the fact that the lymphatic drainage may be irregular, passing by some glands and emptying into more distant ones, or second, by blockage of a main lymphatic trunk, the lymph flow may be turned into by-paths leading into the pre-aortic glands. When the disease is advanced, both of these conditions may be found to exist.

The mortality following resections of the stomach is steadily diminishing; it is now from five to eight per cent., and depends more upon the patient's condition at the time of operation than upon the operation itself. Taking into consideration that, at the present time, the initial recoveries are well above 90 per cent. and that the patients who recover get a chance of radical cure something better than 23 per cent., it certainly seems incumbent upon the medical profession to make a more determined effort toward an early diagnosis.

It may be stated as an axium that cancer as cancer in the stomach does not produce symptoms upon which an early diagnosis can be made; only when its situation makes a palpable tumor-mass or produces obstruction, can a probable diagnosis be established.

*The Small Intestine.* Primary carcinomata of the small intestine are exceedingly rare, and but few cases have come under our observation. Of the 14 cases reported in this series, five were in the duodenum, and none of them were operable. In two cases, the duodeno-jejunal angle was involved; both were inoperable. Of the remaining nine cases, four involved the jejunum, and five the ileum. In two of the five cases submitted to excision, the disease had begun in a pedunculated adenoma or papilloma, and in both intussesception was present.

*The Large Intestine.* The large intestine is a most interesting field for study. Morphologically, it begins at the ileo-cecal orifice and ends at the rectum. Embryologically and physiologically, it is composed of two entirely separate organs. Taking the gastro-intestinal tract as a whole, we find that the celiac axis supplies those organs derived from the foregut, that is, the stomach, liver, pancreas, and duodenum, down to the common duct. The superior mesenteric artery supplies all of those organs which are derived from the midgut and which are instrumental in assimilation and absorption. They are composed of the duodenum beyond the common duct, the jejunum, ileum, cecum, ascending and transverse colon as far as the splenic flexure. Ninety per cent. of the solids are picked up in the small intestine, while 50 per cent. of the liquids and 10 per cent. of the solids are absorbed in the cecum and colon proximal to the splenic flexure. The inferior mesenteric artery supplies the derivatives of the hindgut, and the normal peristalsis, except during defecation, is anti-peristaltic. By means of mucous currents, the distal colon is intermittently engaged in passing material for further elaboration and assimilation backward into the area of midgut absorpton. Under normal conditions the contents of the large intestine, as far as the splenic flexure, are fluid or semi-solid. The descending colon is usually empty, acting as a passage way only into the sigmoid where the feces become more solid in character.

Tumors of the cecum and colon, proximal to the splenic flexure, are sometimes accompanied by changes in metabolism; the nutrition of the patient often suffers and profound anemia may result. Tumors of the descending colon and sigmoid are seldom accompanied by changes in nutrition, and it is the mechanic effects of the tumor which first call attention to the patient's condition.

The surgical treatment of tumors of the large intestine is greatly simplified by the study of its embryology and anatomy. The large intestine is formed on the left side of the abdomen. The head of the colon begins to rotate at about the eleventh week. As the rotation progresses, the colon carries with it its blood vessels, lymphatics, and sympathetic ganglion, and when its normal situation is finally reached, the outer layer of mesenteric attachment is merely a peritoneal adhesion, the division of which, at any situation, enables that part of the colon to be turned upon its long inner mesenteric leaf, which contains all of the important structures.

Generally speaking, malignant disease in any part of the large intestine proximal to the middle of the transverse colon is treated best by removal of all the large intestine up to the growth, doing lateral ileo-colostomy. Beyond this point, resection in continuity, either in a one or two stage operation, is the best plan.

The lymphatics of the large intestine lie with the blood vessels, and usually are easily removed. Many times, when the lymphatics are enlarged, microscopic examination shows the condition to be the result of infection, and not of carcinomata. Proper mobilization of the large intestine, permitting one to secure the blood supply at an early stage of the disease, with careful and accurate removal of the glands, gives a prospect of cure which justifies the most extensive operations. It sometimes happens that one or more loops of the small intestine may have become adherent and involved secondarily with the tumor-mass; as a rule this takes place along the periphery of the bowel. On a number of occasions we have first resected a loop of the small intestine; twice we have removed two separate loops, and in one instance three loops, removing the primary growth in the colon with the portions of the small intestine attached. In four cases we removed a portion of the bladder, and in 3 cases the uterus was coincidently removed. The ultimate results of these cases of multiple resections have been surprisingly good, and we have several such patients alive and well more than five years.

*The Rectum.* The radical cure of carcinomata of the rectum has a bad name in surgery, which is due more to inefficient methods of operation and sentimental attempts to conserve function than to the character and location of the disease. We are coming to the conclusion that a permanent colostomy in the middle of the left rectus muscle should be made as the primary operation in the majority of cases of carcinoma of the ampulla of the rectum, and at a second operation, after the lower segment has been properly cleansed, the entire rectum should be removed from behind. This method offers the patient moderate control, with the best chance of a permanent cure. High rectal and recto-sigmoid growths will often be best approached through the abdomen or by the combined method. The perineal operation should be reserved for growths in the anal region.

The frequency with which secondary carcinomata of the liver, peritoneum, or inoperable glandular involvement occurs makes it imperative in all cases of carcinomata of the rectum and recto-sigmoid first to open and thoroughly explore the abdomen to see whether the case is one which should be submitted to operation and, if advisable, a permanent colostomy can be made at this time.

The diagnosis of cancer of the rectum is so easy that one wonders why so many patients come for consultation without a diagnosis, and why nearly 15 per cent. of them have been recently operated for supposed hemorrhoids. It is a simple matter to examine the rectum digitally, and it requires but little skill to use a proctoscope or sigmoidoscope. Neglect of these simple methods of examination not only causes disaster to the patient, but injury to the practitioner's reputation.

Lack of examination, rather than lack of knowledge, is responsible for most mistakes in diagnosis.

